# Feasibility and Safety of Drug-Coated Balloon for Treatment of *De Novo* Coronary Artery Lesions in Large Vessel Disease: A Large-Scale Multicenter Prospective Study

**DOI:** 10.31083/j.rcm2410277

**Published:** 2023-10-07

**Authors:** Guofeng Gao, Hao-Yu Wang, Yanjun Song, Dong Yin, Lei Feng, Hao Wang, Han Xu, Zhiyong Zhao, Min Yang, Yingming Lu, Zheng Ji, Chenggang Zhu, Kefei Dou

**Affiliations:** ^1^Cardiometabolic Medicine Center, Department of Cardiology, Fuwai Hospital, National Clinical Research Center for Cardiovascular Diseases, National Center for Cardiovascular Diseases, Chinese Academy of Medical Sciences and Peking Union Medical College, 100037 Beijing, China; ^2^State Key Laboratory of Cardiovascular Disease, 100037 Beijing, China; ^3^National Clinical Research Center for Cardiovascular Diseases, 100037 Beijing, China; ^4^Department of Cardiology, XinHua (Chongming) Hospital Affiliated to Shanghai JiaoTong University School of Medicine, 200092 Shanghai, China; ^5^Department of Cardiology, Tangshan Gongren Hospital Affiliated of North China University of Science and Technology, 064012 Tangshan, Hebei, China

**Keywords:** drug-coated balloon, coronary artery disease, percutaneous coronary intervention, large vessel disease

## Abstract

**Background:**

Drug-coated balloons (DCB) have been evaluated to be safe and practical in treating coronary small vessel disease (SVD). However, evidence about the practicality and safety of DCB in treating coronary lesions with diameters greater than 3.0 mm is limited.

**Methods:**

1166 patients who received DCB angioplasty were enrolled and divided into groups of SVD or large vessel disease (LVD) according to the target vessel diameters (<3.0 mm for SVD; ≥3.0 mm for LVD). All participants received a 2-year follow-up. The two main outcomes were patient-oriented composite endpoint (patient-oriented composite endpoint (POCE), all-cause mortality, all myocardial infarctions [MI], or any revascularization), and target lesion failure (target lesion failure (TLF), cardiac death, target vessel MI, or ischemia-driven target lesion revascularization).

**Results:**

In these patients, a total of 30 (2.6%) TLF and 82 (7.0%) POCE were recorded. Patients in the LVD group showed statistically greater rates of lesion success compared to the SVD group (752 [96.0%] vs. 380 [99.2%], *p* = 0.004) and procedural success (751 [95.9%] vs. 380 [99.2%], *p* = 0.003). No significant difference was found in the 2-year risk of TLF (hazard ratio (HR) 1.41, 95% CI 0.58–3.44; *p* = 0.455), POCE (HR 1.29, 95% CI 0.76–2.20; *p* = 0.354), MI (HR 0.88, 95% CI 0.24–3.13; *p* = 0.837), revascularization (HR 1.22, 95% CI 0.68–2.21; *p* = 0.506), and stroke (HR 0.78, 95% CI 0.03–15.26; *p* = 0.784) between the SVD and LVD groups.

**Conclusions:**

There was no discernible inferiority of the DCB intervention in the LVD group as compared to the SVD group. The DCB intervention is practical for coronary lesions with diameters higher than 3.0 mm.

## 1. Introduction

Drug-coated balloon (DCB) is a promising interventional strategy for patients with coronary artery disease (CAD). During percutaneous coronary intervention (PCI), DCB rapidly disperses antiproliferative drugs into the coronary vessel walls via a specialized matrix without permanent metal implantation. Restenosis following percutaneous transluminal coronary angioplasty (PTCA) is dramatically decreased because of the antiproliferative drug coating in DCB and its quick transfer. Numerous DES problems, such as stent thrombosis, are avoided by not permanently implanting stent material. Consequently, the DCB-only approach in PCI represents a cutting-edge substitute for drug-eluting stents (DES) [1].

To date, the feasibility of DCB has been confirmed in in-stent restenosis (ISR) and coronary small vessel disease (SVD) [1, 2, 3]. The application of DCB is generally recommended when there is no flow-limiting dissection or residual stenosis greater than 30% [1, 4]. The use of DCB procedures to treat coronary large vascular disease (LVD, lumen diameter 3.0 mm) has gained popularity in recent years. Recently, by comparing the clinical effectiveness of DCB for LVD with that for SVD, Yu *et al*. [5] undertook a prospective trial to ascertain the viability and safety of DCB in treating CAD patients with *de novo* LVD. The findings revealed no discernible difference in prognosis between patients with LVD and those with SVD, demonstrating the viability of the DCB-only approach in treatments for sizable *de novo* coronary lesions. However, studies with a bigger sample size (>1000) and a longer follow-up (>1 year) are required to verify this finding.

This multiple-center prospective cohort study enrolled 1166 participants treated with DCB and divided them into SVD or LVD groups according to vessel diameter (<3.0 mm for SVD, ≥3.0 mm for LVD), aiming to investigate the feasibility and safety of DCB in treating coronary LVD lesions.

## 2. Methods

### 2.1 Study Design

This multiple-center prospective study enrolled 1166 consecutive patients with CAD who received DCB angioplasty (Bingo paclitaxel-coated) from August 2018 to August 2020 from three teaching hospitals in China (Fuwai hospital, Xinhua (Chongming) hospital, and Tangshan Gongren hospital) with experience in treating patients using DCBs. Eligible patients were those who had *de novo* lesions ≥50% stenosis and were treated with only DCB therapy. Concurrent DES implantation, PCI of in-stent restenosis, and loss to follow-up were the exclusion criteria. There were 9 patients lost to follow-up. SVD and LVD groups of patients were created based on the goal vascular diameter. A coronary lesion with a reference vessel diameter of less than 3.0 mm is referred to as having LVD, whereas one with a reference vessel diameter of more than 3.0 mm is referred to as having SVD. There were 383 patients in the LVD group and 783 in the SVD group. The Institutional Review Boards at the three hospitals gave their approval to the study. This research was conducted after the Helsinki Declaration.

### 2.2 Procedural

All patients received a starting dose of heparin with an initial bolus of 70–100 IU/kg (before the operations) and an extra dose of 1000 IU per hour (during the procedure) along with a dual antiplatelet medication (300 mg of aspirin and 300 mg of clopidogrel the day) prior to the procedure. Either the radial or femoral artery served as the intervention access. Angiography of target vessels with at least two near-orthogonal views after intracoronary nitroglycerin treatment (100–200 µg) was performed in all patients. Before the DCB catheter, lesion preparations by conventional balloon, cutting balloon, non-slip element balloon, and non-compliant balloon were performed, and the target balloon/vessel diameter ratio was 1.0. Successful pre-dilation was defined as no dissections, thrombolysis in myocardial infarction (TIMI) grade 3 flow, and residual stenosis less than 30%. DCB intervention was performed in patients who finished successful pre-dilation. DCB was extended to complete coverage of lesions. DCB/vessel diameter ration and total inflation time were 0.8–1.0 and 30–60 s, respectively. Procedural success was defined as a residual stenosis ≤30% and grade 3 TIMI flow without apparent dissection (of NHLBI65 type C or above).

### 2.3 Data Collection

Data were collected from the electronic medical records of each hospital. Patients’ general data included demographics, history of disease(s), prior operations, and laboratory results. Lesion characteristics included target vessels, types of lesions, lesion length, and diameter stenosis. Diagnosis of hypertension and diabetes was established based on current standard guidelines [6, 7]. The lesion diameter was measured proximal to the lesion.

### 2.4 Follow-Up and Outcomes

After the PCIs, all patients underwent a clinical follow-up that lasted, on average, two years. The clinical follow-up was performed via phone interview or outpatient service every 6 months. A clinical follow-up was given to every participant over the course of a median of two years. Target lesion failure (TLF) and patient-oriented composite endpoint (POCE) are the study’s main outcomes. TLF was outlined as a combination of target vessel myocardial infarctions (MI), target lesion revascularization caused by ischemia, and cardiac death. A composite of all-cause mortality, all MI, or any revascularization is referred to as POCE [8].

### 2.5 Statistical Approaches

The format for categorical variables was “number (%)”. When continuous variables had a regularly distributed distribution, they were referred to as having a “mean ± standard deviation (SD)”. When continuous variables were regularly distributed, the two-sample *t*-test was employed to determine if there were statistical differences; otherwise, the Mann-Whitney U test was applied. The use of the χ^2^ test was used to examine categorical variable differences. Kaplan–Meier (KM) plots for comparing the risk of outcomes between SVD and LVD were drawn to visualize differences in endpoints. Multiple-Cox regression analysis was conducted to confirm the difference in the risk of clinical outcomes between the SVD and LVD groups. Adjusted cofounding factors include age, sex, current smoking, body mass index (BMI), hypertension, diabetes mellitus, chronic kidney disease, prior MI, prior PCI, prior coronary artery bypass grafting (CABG), acute coronary syndrome (ACS), thrombotic lesion, chronic total occlusion, lesion length, dissection after DCB treatment, procedural success, and intravascular ultrasound (IVUS) use. The cut-off for statistical significance was two-sided less than 0.05. We used R Studio software version 4.1.3 (R Foundation for Statistical Computing, Vienna, Austria) for all statistical analyses.

## 3. Results

### 3.1 Baseline Characteristics

Of these patients, 843 (72.3%) were males and the mean age was 61.55 ± 12.46 years. There were ten procedures performed via the femoral artery and the rest of the procedures were performed via the radial approach. Patients in the LVD group had a lower incidence of hypertension (532 [67.9%] vs. 216 [56.4%], *p* < 0.001), diabetes (299 [38.2%] vs. 101 [26.4%], *p* < 0.001) and dyslipidemia (413 [52.7%] vs. 114 [29.8%], *p* < 0.001). The incidence of ACS was significantly higher in the LVD group (583 [74.5%] vs. 312 [81.5%], *p* = 0.010) (Table 1).

**Table 1.** **Baseline patient characteristics**.

		All	SVD group (<3.0 mm)	LVD group (≥3.0 mm)	*p* value
		(n = 1166)	(n = 783)	(n = 383)	
Age		61.55 ± 12.46	61.99 ± 12.28	60.66 ± 12.78	0.086
Male		843 (72.3)	565 (72.2)	278 (72.6)	0.934
BMI		25.55 ± 3.48	25.58 ± 3.38	25.47 ± 3.68	0.603
Diabetes		400 (34.3)	299 (38.2)	101 (26.4)	<0.001
Hypertension		748 (64.2)	532 (67.9)	216 (56.4)	<0.001
Dyslipidemia		527 (45.2)	413 (52.7)	114 (29.8)	<0.001
Chronic kidney disease		209 (17.9)	152 (19.4)	57 (14.9)	0.07
Smoking		315 (27.0)	201 (25.7)	114 (29.8)	0.159
Atrial fibrillation		56 (4.8)	36 (4.6)	20 (5.2)	0.747
Heart failure		37 (3.2)	24 (3.1)	13 (3.4)	0.902
Prior MI		227 (19.5)	164 (20.9)	63 (16.4)	0.081
Prior PCI		356 (30.5)	263 (33.6)	93 (24.3)	0.002
Prior CABG		24 (2.1)	17 (2.2)	7 (1.8)	0.866
Prior stroke		89 (7.6)	48 (6.1)	41 (10.7)	0.008
Peripheral vascular disease		46 (3.6)	30 (3.8)	12 (3.1)	0.665
Previous bleeding		35 (3.0)	31 (4.0)	4 (1.0)	0.006
Clinical presentation					
Stable CAD		272 (23.3)	200 (25.5)	72 (18.8)	0.013
Acute coronary syndrome		895 (76.8)	583 (74.5)	312 (81.5)	0.01
	Unstable angina	738 (63.3)	258 (67.4)	480 (61.3)	
	NSTEMI	84 (7.2)	31 (8.1)	53 (6.8)	
	STEMI	72 (6.2)	22 (5.7)	50 (6.4)	
Creatinine clearance, mL/min		88.07 ± 34.62	85.97 ± 35.36	92.35 ± 32.68	0.003
NT-proBNP		316.94 ± 490.62	320.66 ± 522.89	309.34 ± 417.50	0.712
Hemoglobin, g/L		113.60 ± 15.17	132.63 ± 14.61	135.58 ± 16.09	0.002
Platelet, ×${\mathrm{10}}^{9}$/L		217.60 ± 55.78	218.09 ± 55.83	216.60 ± 55.75	0.669
HbA1c		6.68 ± 1.25	6.70 ± 1.28	6.63 ± 1.18	0.348
Anticoagulation therapy		70 (6.0)	46 (5.9)	24 (6.3)	0.894

Note: All data are presented as n (%) or mean ± SD. Abbreviations: SVD, small vessel disease; LVD, large vessel disease; BMI, body mass index; MI, myocardial infarction; PCI, percutaneous coronary intervention; CABG, coronary artery bypass grafting; CAD, coronary artery disease; NSTEMI, non-ST segment elevation myocardial infarction; STEMI, ST segment elevation myocardial infarction; NT-proBNP, n-terminal pro-brain natriuretic peptide; HbA1c, glycosylated hemoglobin A1c.

The target lesion in the LVD group was mainly located in the left anterior descending artery (LAD, 225 [58.7%]) and right coronary artery (RCA, 82 [21.1%]). The left circumflex artery (247 [31.5%]) was the main targeted vessel in the SVD group. Compared with the SVD group, patients with LVD had lower rates of bifurcation lesions (114 [14.6%] vs. 38 [9.9%], *p* = 0.034), calcified lesions (288 [36.8%] vs. 101 [26.4%], *p* = 0.001), chronic total occlusions (86 [11.0%] vs. 26 [6.8%], *p* < 0.001) and American College of Cardiology/American Heart Association (ACC/AHA) type B2/C lesions (334 [42.7%] vs. 79 [20.6%], *p* < 0.001), and a smaller incidence of diameter stenosis (88.72 ± 8.53 vs. 84.70 ± 9.56, *p* < 0.001) (Table 2).

**Table 2.** **Lesion characteristics**.

		All	SVD group (<3.0 mm)	LVD group (≥3.0 mm)	*p* value
		(n = 1166)	(n = 783)	(n = 383)	
Target vessel					
	Left main	4 (0.3)	1 (0.1)	3 (0.8)	0.206
	Left anterior descending artery	416 (35.7)	191 (24.4)	225 (58.7)	<0.001
	Diagonal branch	133 (11.4)	113 (14.4)	20 (5.2)	<0.001
	Left circumflex artery	308 (26.4)	247 (31.5)	61 (15.9)	<0.001
	Obtuse marginal branch/ramus	83 (7.1)	75 (9.6)	8 (2.1)	<0.001
	Right coronary artery	165 (14.2)	84 (10.7)	81 (21.1)	<0.001
	PDA/PL	96 (8.2)	91 (11.6)	5 (1.3)	<0.001
	Graft	7 (0.6)	5 (0.6)	2 (0.5)	1.00
Bifurcation lesion		152 (13.0)	114 (14.6)	38 (9.9)	0.034
Calcified lesion		389 (33.4)	288 (36.8)	101 (26.4)	0.001
CTO		112 (9.6)	86 (11.0)	26 (6.8)	0.029
Thrombus lesion		27 (2.3)	18 (2.3)	9 (2.3)	1.00
Reference vessel diameter by visual estimation, mm		2.65 ± 0.54	2.34 ± 0.30	3.29 ± 0.33	<0.001
Lesion length, mm		18.66 ± 10.30	18.01 ± 9.86	19.99 ± 11.03	0.002
Diameter stenosis, %		87.40 ± 9.08	88.72 ± 8.53	84.70 ± 9.56	<0.001
ACC/AHA type B2/C lesions		413 (35.4)	334 (42.7)	79 (20.6)	<0.001

Note: All data are presented as n (%) or mean ± SD. Abbreviations: SVD, small vessel disease; LVD, large vessel disease; PDA/PL, posterior descending artery/posterior branch of the left ventricle; CTO, chronic total occlusion; ACC/AHA, American College of Cardiology/American Heart Association.

### 3.2 Procedural Comparisons

In the interventional procedure, the percentages of using a cutting balloon (68 [8.7%] vs. 70 [18.3%], *p* < 0.001), non-slip element balloon (156 [19.9%] vs. 141 [36.8%], *p* < 0.001) and non-compliant balloon (180 [23.0%] vs. 160 [41.8%], *p* < 0.001) were higher in the LVD group compared with the SVD group. The mean diameter (2.31 ± 0.30 vs. 3.17 ± 0.38, *p* < 0.001), mean number (1.09 ± 0.31 vs. 1.25 ± 0.49, *p* < 0.001), and total length of DCB (21.84 ± 10.11 vs. 28.58 ± 16.42, *p* < 0.001) used in the LVD group were higher than that of the SVD group. Device success, lesion success, and procedural success were achieved in most patients in both groups, whereas the rates of lesion success (752 [96.0%] vs. 380 [99.2%], *p* = 0.004) and procedural success (751 [95.9%] vs. 380 [99.2%], *p* = 0.003) in LVD group were statistically higher than that of the SVD group (Table 3). The rate of procedural complications in the LVD group was significantly lower than in the SVD group (37 [4.7%] vs. 8 [2.1%], *p* = 0.028).

**Table 3.** **Procedural characteristics**.

		All	SVD group (<3.0 mm)	LVD group (≥3.0 mm)	*p* value
		(n = 1166)	(n = 783)	(n = 383)	
Balloon pre-dilation		1157 (98.9)	772 (98.6)	381 (99.4)	0.187
Mean diameter of pre-dilation balloon, mm		2.02 ± 0.38	1.96 ± 0.35	2.18 ± 0.42	<0.001
Mean length of pre-dilation balloon, mm		15.25 ± 2.57	14.89 ± 2.34	16.25 ± 2.89	<0.001
Mean inﬂation pressure with pre-dilation balloon		13.02 ± 3.45	12.47 ± 3.31	14.53 ± 3.35	<0.001
Cutting balloon		138 (11.8)	68 (8.7)	70 (18.3)	<0.001
Non-slip element balloon		297 (25.5)	156 (19.9)	141 (36.8)	<0.001
Non-compliant balloon		340 (29.2)	180 (23.0)	160 (41.8)	<0.001
Rotational atherectomy		5 (0.4)	3 (0.4)	2 (0.5)	0.666
DCB					
	Mean diameter, mm	2.60 ± 0.52	2.31 ± 0.30	3.17 ± 0.38	<0.001
	Mean number	1.14 ± 0.38	1.09 ± 0.31	1.25 ± 0.49	<0.001
	Total length, mm	24.06 ± 12.92	21.84 ± 10.11	28.58 ± 16.42	<0.001
	Mean inﬂation pressure, atm	9.28 ± 2.85	9.20 ± 2.99	9.46 ± 2.54	0.147
	Mean duration of inﬂation, s	55.67 ± 12.14	55.46 ± 13.46	56.10 ± 8.87	0.398
Dissection after DCB treatment		40 (3.4)	32 (4.1)	8 (2.1)	0.112
Bail-out stenting		21 (1.8)	14 (1.8)	7 (1.8)	1.00
IVUS		109 (9.3)	29 (3.7)	80 (20.9)	<0.001
Periprocedural complications		45 (3.9)	37 (4.7)	8 (2.1)	0.028
Device success		1145 (98.2)	769 (98.2)	376 (98.2)	1.00
Lesion success		1132 (97.1)	752 (96.0)	380 (99.2)	0.004
Procedural success		1131 (97.0)	751 (95.9)	380 (99.2)	0.003

Note: All data are presented as n (%) or mean ± SD. Abbreviations: SVD, small vessel disease; LVD, large vessel disease; DCB, drug-coated balloon; IVUS, intravenous-ultrasound.

### 3.3 DCB for Lesions in LVD

**Fig. 1.**
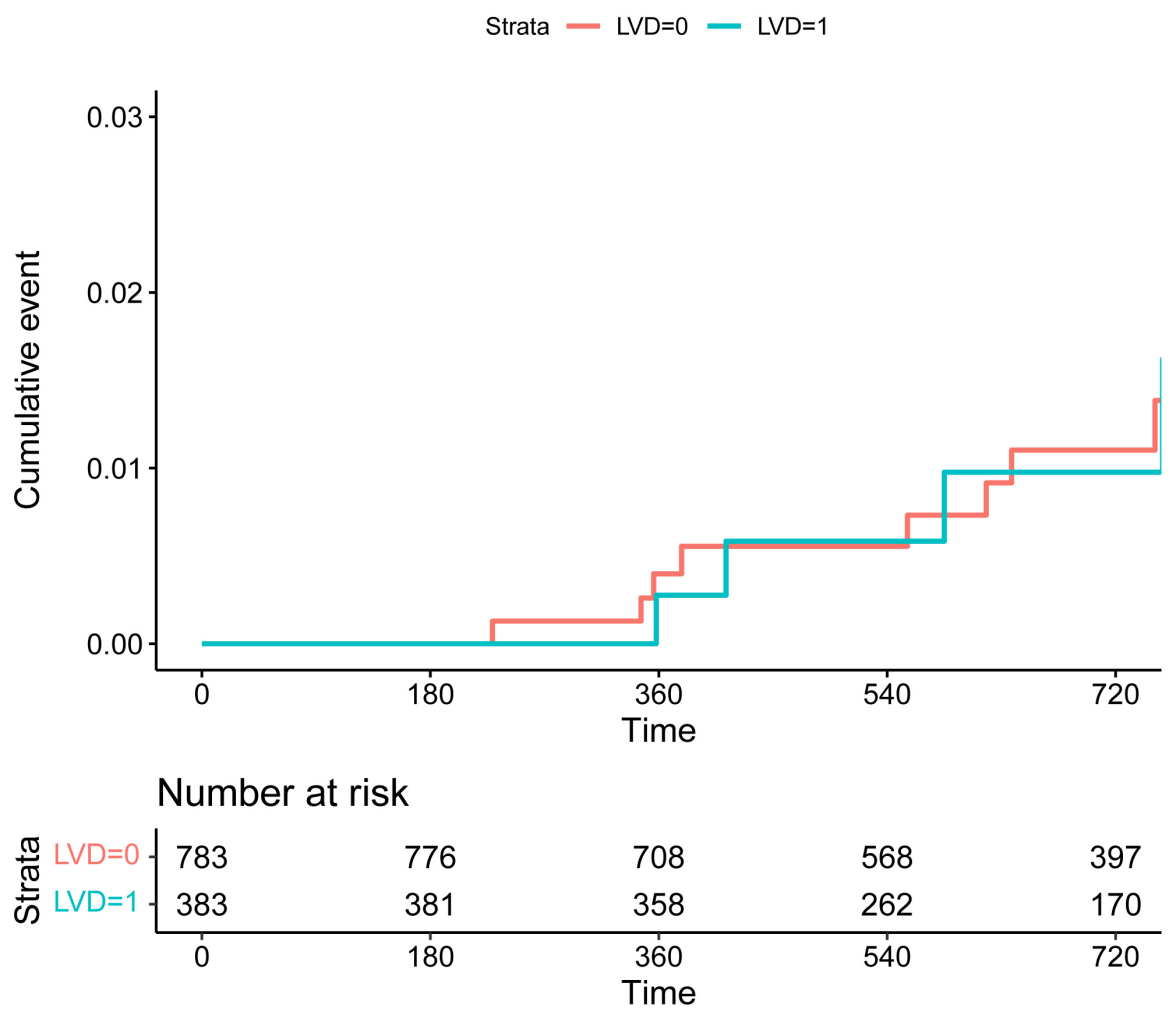
**Kaplan-Meier curves of the risk of MI for patients in the groups of SVD (LVD = 0) and LVD (LVD = 1). **Abbreviations: MI, myocardial infarction; SVD, small vessel disease; LVD, large vessel disease.

**Fig. 2.**
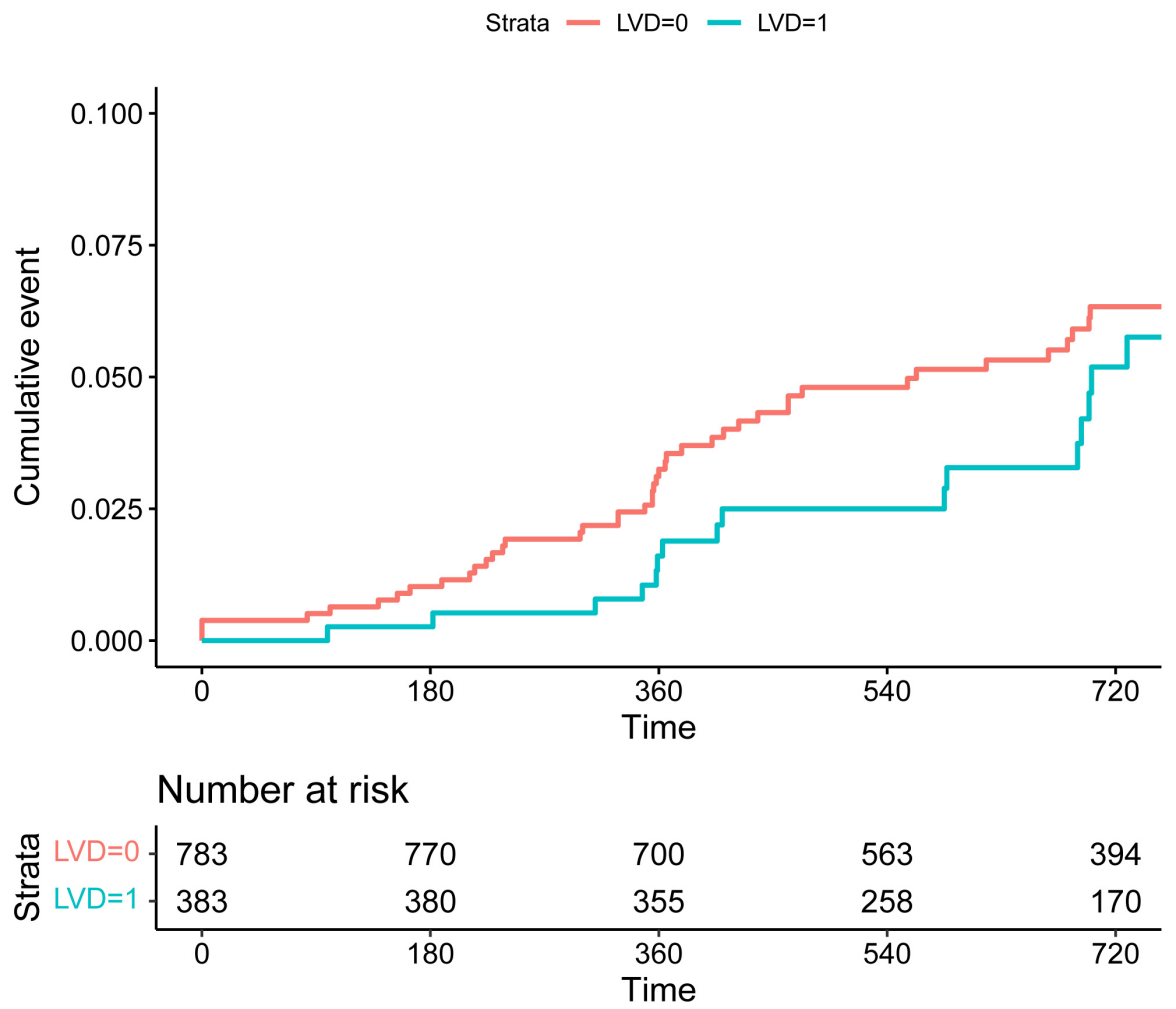
**Kaplan-Meier curves of the risk of POCE for patients in the groups of SVD (LVD = 0) and LVD (LVD = 1).** Abbreviations: POCE, patient-oriented composite endpoint; SVD, small vessel disease; LVD, large vessel disease.

**Fig. 3.**
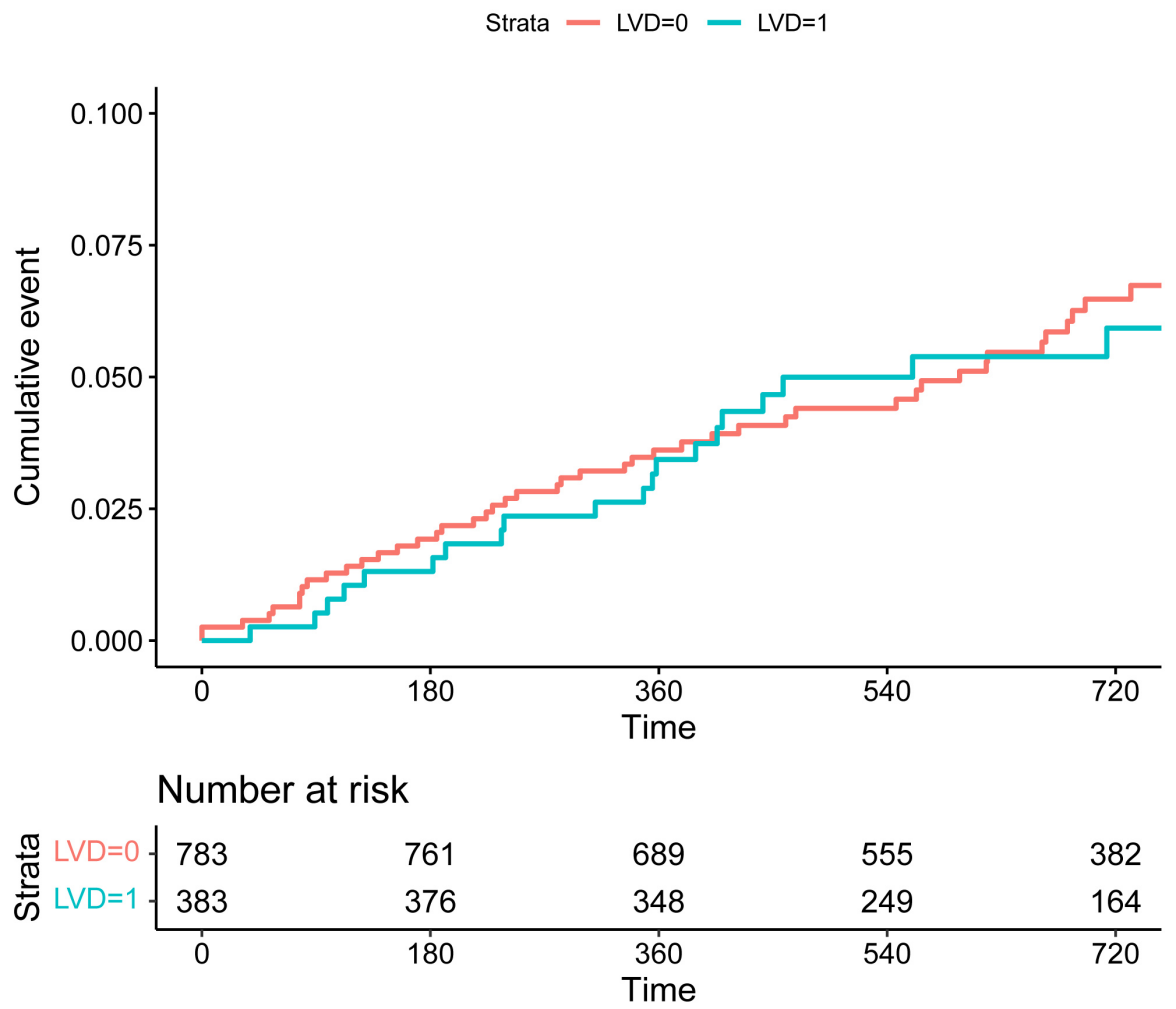
**Kaplan-Meier curves of the risk of revascularization for patients in the groups of SVD (LVD = 0) and LVD (LVD = 1).** Abbreviations: SVD, small vessel disease; LVD, large vessel disease.

**Fig. 4.**
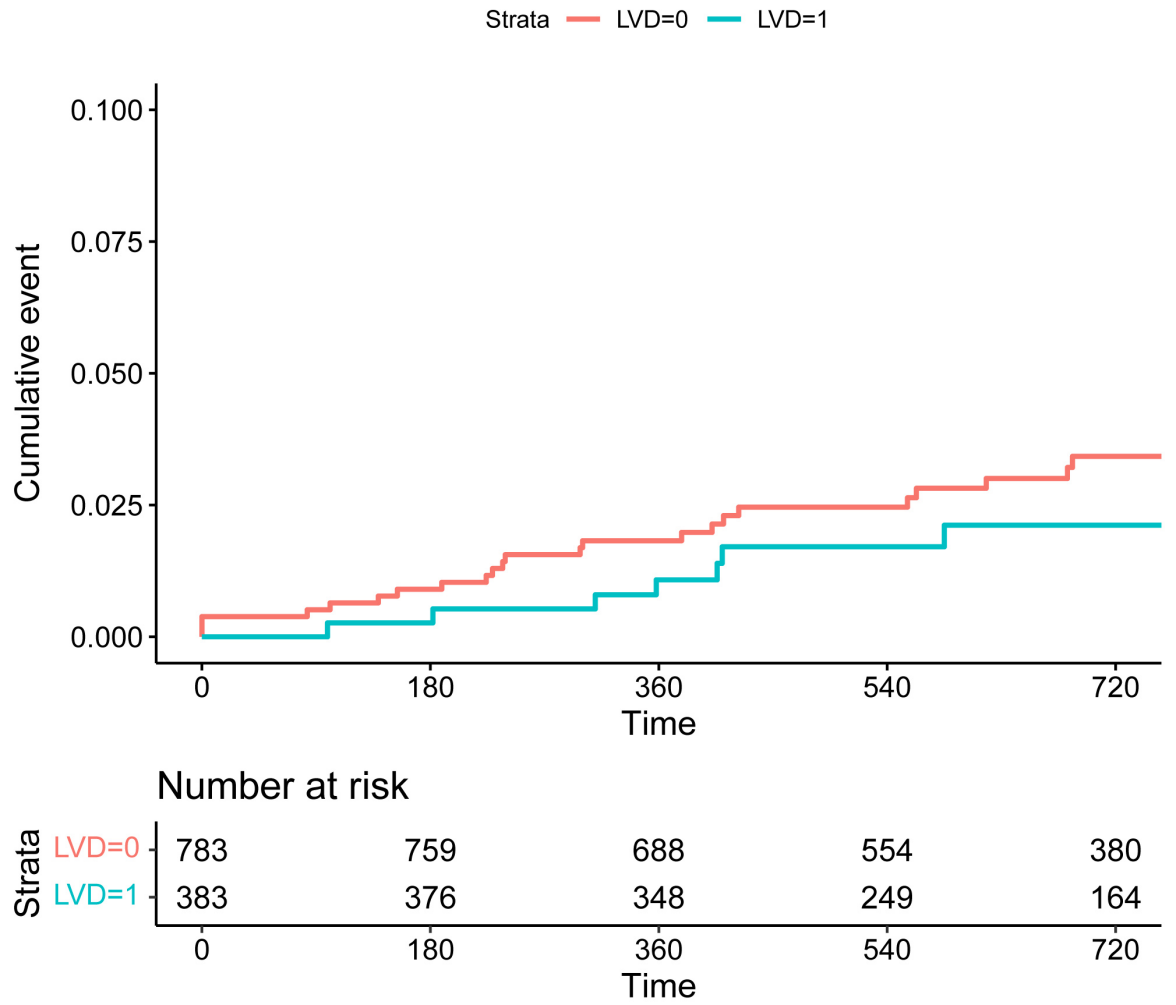
**Kaplan-Meier curves of the risk of TLF for patients in the groups of SVD (LVD = 0) and LVD (LVD = 1).** Abbreviations: TLF, target lesion failure; SVD, small vessel disease; LVD, large vessel disease.

After the DCB intervention, only a small number of patients developed clinical events such as TLF (30 [2.6%]), MI (12 [1.0%]), revascularization (65 [5.6%]), stroke (10 [0.9%]) and all-cause death (11 [0.9%]). In comparisons between two groups, there was no statistical difference in the LVD group in the risk of clinical outcomes in the model with adjustment of age, sex, current smoking, BMI, hypertension, diabetes mellitus, chronic kidney disease, prior MI, prior PCI, prior CABG, ACS, thrombotic lesion, chronic total occlusion, lesion length, dissection after DCB treatment, procedural success, and IVUS use (Table 4) (TLF: hazard ratio (HR) 1.41, 95% CI 0.58–3.44, *p* = 0.455; POCE: HR 1.29, 95% CI 0.76–2.20, *p* = 0.354; MI: HR 0.88, 95% CI 0.24–3.13, *p* = 0.837; revascularization: HR 1.22, 95% CI 0.68–2.21, *p* = 0.506; stroke: HR 0.78, 95% CI 0.03–15.26, *p* = 0.784). The KM plots revealed no significant difference in the risk of composite endpoint of TLF and POCE, as well as MI and revascularization between the two groups (Figs. 1,2,3,4, **Supplementary Table 1**).

**Table 4.** **2-year clinical outcomes stratified by the presence of small vessel disease**.

		All	SVD group (<3.0 mm)	LVD group (≥3.0 mm)	Unadjusted HR (95% CI)	*p* value*	Adjusted HR (95% CI)	*p* ${\mathrm{value}}^{\#}$
		(n = 1166)	(n = 783)	(n = 383)				
Target lesion failure†		30 (2.6)	23 (2.9)	7 (1.8)	1.61 (0.69 to 3.75)	0.265	1.41 (0.58 to 3.44)	0.455
Patient-oriented composite endpoint††		82 (7.0)	59 (7.5)	23 (6.0)	1.24 (0.77 to 2.01)	0.376	1.29 (0.76 to 2.20)	0.354
All-cause death		11 (0.9)	10 (1.3)	1 (0.3)	4.84 (0.62 to 37.84)	0.096	2.20 (0.26 to 18.79)	0.473
	Cardiac death	4 (0.3)	4 (0.5)	0 (0.0)	NA	NA	NA	NA
Myocardial infarction		12 (1.0)	8 (1.0)	4 (1.0)	0.97 (0.29 to 3.22)	0.958	0.88 (0.24 to 3.13)	0.837
	Target vessel MI	10 (0.9)	7 (0.9)	3 (0.8)	1.14 (0.29 to 4.40)	0.852	1.05 (0.25 to 4.44)	0.948
Any revascularization		65 (5.6)	45 (5.7)	20 (5.2)	1.09 (0.64 to 1.85)	0.832	1.22 (0.68 to 2.21)	0.506
	Ischemia-driven TVR	33 (2.8)	21 (2.7)	12 (3.1)	0.84 (0.42 to 1.71)	0.637	0.80 (0.37 to 1.73)	0.563
	Ischemia-driven TLR	18 (1.5)	12 (1.5)	6 (1.6)	0.98 (0.37 to 2.61)	0.968	0.80 (0.29 to 2.25)	0.674
Stroke		10 (0.9)	7 (0.9)	3 (0.8)	0.97 (0.25 to 3.78)	0.969	0.78 (0.03 to 15.26)	0.784

The median follow-up duration was 2.0 years (interquartile range: 1.3 to 2.5). *p* value*: *p* value for the unadjusted model; *p* ${\mathrm{value}}^{\#}$: *p* value for the adjusted model. †Target lesion failure was defined as a composite of cardiac death, target vessel MI, or ischemia-driven TLR. ††Patient-oriented composite endpoint was defined as a composite of all-cause death, all MI, or any revascularization. Model adjusted for age, sex, current smoking, body mass index, hypertension, diabetes mellitus, chronic kidney disease, prior MI, prior PCI, prior CABG, acute coronary syndrome, thrombotic lesion, chronic total occlusion, lesion length, dissection after DCB treatment, procedural success, and IVUS use. Abbreviations: SVD, small vessel disease; LVD, large vessel disease; HR, hazard ratio; MI, myocardial infarction; TVR, target vessel revascularization; TLR, target lesion revascularization; PCI, percutaneous coronary intervention; CABG, coronary artery bypass grafting; DCB, drug-coated balloon; IVUS, intravenous-ultrasound; NA, not applicable.

## 4. Discussion

In this multi-center study, we enrolled 1166 patients who received DCB treatment and evaluated the application of the DCB-only strategy in treating *de novo* coronary lesions in LVD. Two conclusions can be drawn from our study: (1) DCB intervention is safe and practical in treating LVD lesions; (2) the risk of clinical event rates (TLF and POCE) in patients with LVD was similar to that of patients with SVD after DCB treatment. These results indicate that DCB-only treatment is safe and efficient for large *de novo* coronary lesions with diameters ≥3.0 mm.

DCB, a novel therapeutic strategy based on the rapid and homogenous transfer of anti-proliferative drugs from the balloon into the vessel wall without any remaining permanent stents, has been widely reported for its great clinical value in treating CAD. In a recent international DCB consensus, DCB-only percutaneous coronary intervention was thought to be a promising concept for treating coronary lesions and is recommended to be used in every lesion for any PCI [1].

DCB is increasingly regarded as a DES alternative option for various clinical situations. ISR lesion was the first lesion targeted by DCB. In 2018, Jensen *et al*. [9] performed a multicenter randomized control trial (RCT) that enrolled 229 patients with ISR in bare metal stents (BMS) or DES. Researchers observed that the rate of TLF between the DCB and DES groups was not significantly different and that the 6-month late lumen loss (LLL) in the DCB group was comparable to the DES group (*p* = 0.40). Similar conclusions were also reported by Unverdorben *et al*. [10]. Besides, several RCTs also focused on the effectiveness of DCB treatment for SVD lesions [3, 11, 12]. Specifically, in the BASKET-SMALL 2 study, 758 patients with *de novo* lesions in small coronary vessels were enrolled and randomly divided into the groups of DCB or DES. Scholars reported that DCB showed its similar efficacy and safety for SVD, as investigators found that LLLs were similar in the two groups and the risk of MACEs is also similar between the DCB and DES groups [3]. These results were also supported by several RCTs such as PICCOLETO [12] and the RESTORE SVD study [11], demonstrating the increased therapeutic value of DCB of treating coronary lesions in small vessels. In addition, recent research conducted in the PICCOLETO II study has reported the 3-year clinical outcomes, demonstrating a higher risk of major adverse cardiac events (MACEs) in patients with *de novo* lesions in SVD who were treated with DES compared to those treated with new-generation paclitaxel drug-coated balloons (DCB) alone. These findings provide further support for the feasibility and safety of DCB-only treatment and suggest a potential superiority of DCB in the management of SVD lesions [13]. Besides, DCB was further recommended in patients with diabetes mellitus [14], acute coronary syndrome [15, 16, 17], and high bleeding risk (HBR) [18].

Previous data concerning the therapeutic roles of DCB in LVD is scarce. Compared with small vessels, large coronary vessels have more smooth muscle fibers, which are more susceptible to vascular dissection or recoil, leading to adverse cardiac events such as acute occlusion or restenosis. For this reason, cardiologists still held doubts about the safety and practicality of the DCB-only intervention for *de novo* coronary lesions with large lumens. However, our study effectively addressed and alleviated this concern, as the rate of procedural success in the LVD group was statistically higher than that of the SVD group (LVD vs. SVD: 99.2% vs. 95.9%, *p* = 0.003), and the incidence of dissection after DCB was lower in the LVD group than that in the SVD group although there was no statistical difference between the groups (LVD vs. SVD: 2.1% vs. 4.1%, *p* = 0.112). Similar results were also reported by Yu *et al*. [5]. Similar to our research, their results showed increased safety of DCB intervention in treating LVD, as all participants with LVD received successful procedures and the rate of dissection after DCB intervention was lower than that in the SVD group (LVD vs. SVD: 28.3% vs. 35.7%, *p* = 0.112). The DCB-alone intervention in LVD also showed similar efficiency in the long-term clinical risk when compared with the SVD group. In addition to clinical events, scholars also showed that there was no significant difference between the SVD and LVD groups in LLL [19]. Similar results were also found in previous studies whose exclusion criteria did not include patients with LVD [16, 20]. These studies demonstrated that DCB-alone intervention is practical and safe in treating coronary large vessel lesions, however, it still lacks randomized data or prospective cohort studies, which will need to be performed.

Short-term dual antiplatelet therapy (DAPT) is an additional advantage of DCB for PCI. Basket small 2 sub-analysis focused on HBR patients and found that rates of major bleeding events were lower in patients treated with DCB when compared with patients treated with DES as the DCB groups received shorter DAPT treatment [3]. The therapeutic potential of DCB treatment in HBR patients was further demonstrated in the DEBUT study [20]. In addition, a low risk of vessel thrombosis with the DCB-only intervention was also determined by previous studies, even with only 1 month of DAPT [19, 21]. DAPT regimens for patients treated with DCB have not been formally recommended in guidelines. In our study, a 4-week DAPT was given to all patients, which was recommended based on expert opinion [1]. Specific numbers of bleeding or thrombotic events are not presented in our study, but there was no fatal case reported in our cohort. DCBs clinical value for HBR patients with CAD should be further investigated. In addition to DAPT, studies revealed that P2Y12 inhibitor monotherapy following 1–3 months DAPT yields comparable rates of adverse cardiac events and a reduced risk of major bleeding, irrespective of the complexity of the PCI. This suggests that P2Y12 inhibitor monotherapy might be a better anti-platelet therapy in patients undergoing PCI [22]. However, the full potential and value of P2Y12 inhibitor monotherapy has yet to be thoroughly investigated, and further studies are warranted to explore its clinical implications.

Several factors were found to be associated with an increased risk of poor prognosis. A recent individual patient data meta-analysis by De Luca *et al*. [23] demonstrated that severe acute respiratory syndrome coronavirus 2 (SARS-CoV-2) infection was independently linked to a higher risk of in-hospital death among patients undergoing PCI. Although our study included 373 patients during the COVID-19 pandemic, none of them were diagnosed with the SARS-CoV-2 infection, indicating no direct impact on the prognosis of these patients. However, given the potential risk factor that SARS-CoV-2 poses for poor prognosis in coronary heart disease, it should be given considerable attention, especially in the post-pandemic era.

The relationship between operator experience, procedural volume and clinical outcomes has also been extensively studied for PCI, and the operator’s procedural volume has been identified as a key factor influencing prognosis [24, 25]. Studies have demonstrated that PCI patients treated by operators with a high procedural volume (>557 cases per year) exhibit a significantly lower rate of periprocedural mortality compared to those treated by operators with a lower procedural volume [26]. Although Fuwai hospital is large-volume single center with experienced, high-volume operators of PCI procedures [27], it should be noted that we did not specifically record the annual operator PCI case volume of each center in our study. Exploring the effect of operator experience on the prognosis of patients undergoing DCB-only intervention would be interesting. Additionally, the timing of the procedure has been suggested to affect patient prognosis. Studies have indicated that PCIs performed during off-hours might be associated with increased periprocedural mortality compared to those performed during regular working hours [28]. However, we did not record the specific time when the procedure was performed. Investigating the association between the timing of DCB-only PCIs and cardiovascular risk in patients will need to be investigated. Overall, these factors, including SARS-CoV-2 infection, operator experience/procedural volume and procedure timing, can play a significant role in the prognosis of patients undergoing DCB-only interventions. Further research exploring their impacts will provide valuable insights into optimizing patient outcomes.

There are several limitations in the current study. First, as a real-world multi-center trial in a referral university hospital with complex PCI knowledge [29, 30], the operating capacities of DCB intervention in different centers are varied, which may influence the procedural and follow-up outcomes. Second, the outcomes of this study only focused on clinical events, but no anatomic outcomes were recorded. The lack of concrete conditions of target vessels described by LLL might weaken the level of evidence. Finally, this was not a randomized study. Further randomized and prospective studies are required to validate these conclusions.

## 5. Conclusions

This multicenter, prospective cohort study showed that the application of paclitaxel DCB alone in treating large coronary vessel disease is as safe and effective as that for small coronary vessel disease, with similar clinical events with a median follow-up of 2 years. Studies comparing the efficiency of DCB and DES in treating LVD in patients with CAD, and related RCT are necessary to further clarify the clinical role of DCB.

## Data Availability

The datasets used and/or analyzed during the current study are available from the corresponding author on reasonable request.

## Supplementary Material

Supplementary material associated with this article can be found, in the online version, at https://doi.org/10.31083/j.rcm2410277.
