# Supplementary material for: Feasibility and Safety of Drug-Coated Balloon for Treatment of De Novo Coronary Artery Lesions in Large Vessel Disease: A Large-Scale Multicenter Prospective Study
Source: Rev Cardiovasc Med. 2023 Oct 7;24(10):277. doi: 10.31083/j.rcm2410277 (PMC11273142; doi:10.31083/j.rcm2410277)
Supplement: Supplementary file 1 [file 2153-8174-24-10-277-s1.docx]

**Supplementary Materials**

**Feasibility and Safety of Drug-coated Balloon for treatment of de-novo coronary artery lesions in large vessel disease: A Large-scale Multicenter Prospective Study**

Supplementary Table 1. 2-year clinical outcomes stratified by the presence of small or large vessel disease

|  | All  (n=1166) | SVD group (<3.0mm)  (n=783) | LVD group (≥3.0mm)  (n=383) | Difference  (95% CI) | P value* |
| --- | --- | --- | --- | --- | --- |
| Target lesion failure† | 30 (2.6) | 23 (2.9) | 7 (1.8) | 1.11 (-0.68 to 2.90) | 0.265 |
| Patient-oriented composite endpoint†† | 82 (7.0) | 59 (7.5) | 23 (6.0) | 1.53 (-1.48 to 4.54) | 0.376 |
| All-cause death | 11 (0.9) | 10 (1.3) | 1 (0.3) | 1.02 (-0.03 to 2.10) | 0.096 |
| Cardiac death | 4 (0.3) | 4 (0.5) | 0 (0.0) | 0.51 (-0.05 to 1.31) | 0.159 |
| Myocardial infarction | 12 (1.0) | 8 (1.0) | 4 (1.0) | -0.02 (-1.26 to 1.22) | 0.958 |
| Target vessel MI | 10 (0.9) | 7 (0.9) | 3 (0.8) | 0.11 (-0.99 to 1.21) | 0.852 |
| Any revascularization | 65 (5.6) | 45 (5.7) | 20 (5.2) | 0.53 (-2.24 to 3.29) | 0.832 |
| Ischemia-driven TVR | 33 (2.8) | 21 (2.7) | 12 (3.1) | -0.45 (-2.53 to 1.63) | 0.637 |
| Ischemia-driven TLR | 18 (1.5) | 12 (1.5) | 6 (1.6) | -0.03 (-1.55 to 1.48) | 0.968 |
| Stroke | 10 (0.9) | 7 (0.9) | 3 (0.8) | 0.11 (-0.99 to 1.21) | 0.969 |

The median follow-up duration was 2.0 years (interquartile range: 1.3 to 2.5).

*A log-rank test was used to calculate P values.

†Target lesion failure was deﬁned as a composite of cardiac death, target vessel MI, or ischemia-driven TLR. ††Patient-oriented composite endpoint was deﬁned as a composite of all-cause death, all MI, or any revascularization.

Abbreviations: MI, myocardial infarction; TVR, target vessel revascularization; TLR, target lesion revascularization.
